# Twinkle Artifact Observed During POCUS of a Human Myiasis Caused by the *Dermatobia hominis*Botfly

**DOI:** 10.24908/pocus.v8i2.16712

**Published:** 2023-11-27

**Authors:** David Jerome, Matthew Stacey, Joseph Newbigging

**Affiliations:** 1 Northern Ontario School of Medicine University Thunder Bay, ON Canada; 2 Department of Medicine, Queen's University Kingston, ON Canada

**Keywords:** Point of Care Ultrasound(POCUS), Emergency Medicine, wilderness medicine, travel medicine, infectious disease

## Abstract

An 81-year-old man presented to urgent care for assessment of an area of erythema and tenderness on his right thigh after recent travel to Belize. Point of care ultrasound (POCUS) revealed a hyperechoic structure with acoustic shadowing in the subcutaneous tissue. Colour Doppler assessment of the structure produced a twinkle artifact. The structure was removed and pathology identified the object as a *Dermatobia hominis* larva (human botfly). The use of POCUS helped identify and localize the subcutaneous foreign body. The use of colour Doppler produced the twinkle artifact, which has not been previously reported as a finding produced during ultrasonographic assessment of botfly larvae.

## Introduction

Myiasis is the infestation of live human and vertebrate animal tissues by botfly larvae. A number of botfly species are capable of causing human myiasis. The most common form of myiasis encountered in North America is due to infestation by *Dermatobia hominis (D. hominis)* in travellers returning from Central and South America 1. The *D. hominis* fly lays her eggs on the underside of a blood-sucking arthropod (usually a mosquito). When the arthropod host subsequently feeds on a human, the botfly eggs hatch and the larvae infiltrate into the human’s tissues 1, 2. Once in the human host, botfly larvae live in the subcutaneous tissue and feed on the host’s tissues until the larvae are mature and spontaneously exit the host. Human infestation usually lasts for four to eighteen weeks. 

Patients with a botfly infestation present with an erythematous, raised, furuncle-like lesion 1, 2, 3, 4. The lesion usually appears to have central umbilication or necrosis. Patients sometimes report the sensation of movement within the subcutaneous tissues. Larval removal can be facilitated by the application of an occlusive substance over the wound (such as petroleum jelly). This starves the larva of oxygen, causing it to emerge from the host’s skin 1. 

Here we present a case report of the use of point of care ultrasound (POCUS) to assess a patient with myiasis, including the first known report of a Doppler twinkle artifact observed during ultrasound assessment of a human myiasis.

## Case Report

An 81-year-old man presented to an urgent care centre in Ontario, Canada for assessment of an area of swelling and erythema on his right thigh. The patient was bitten by an unknown insect five weeks previous while travelling in Belize and his symptoms developed around the site of the insect bite. He had presented to a primary care provider two weeks prior for assessment of the lesion where he was diagnosed with cellulitis and prescribed a course of oral cephalexin. The patient reported that his symptoms initially improved while taking the antibiotics but reoccurred after the course of antibiotics was complete. The patient denied any recent history of fevers, chills, nausea, vomiting or diarrhea. He did not have any calf swelling, calf pain, dyspnea or hemoptysis.

On presentation, the patient’s vital signs were within normal limits. He had a round, raised, 5-10 millimetre-wide area of inflammation on the medial aspect of his right thigh, resembling a furuncle. There was no fluctuance, fluid drainage, or visible break in the skin. 

**Figure 1:**
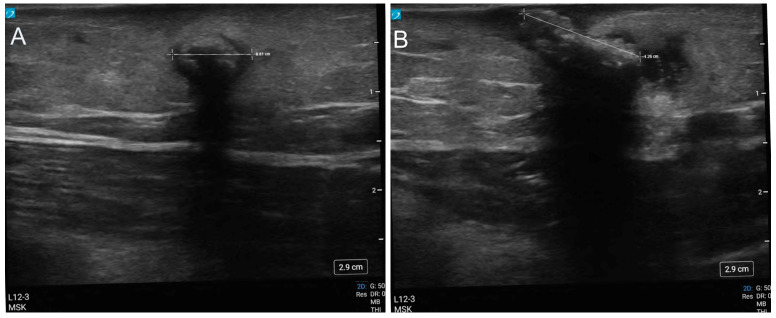
POCUS images of the myiasis viewed in short axis (A), and in long axis (B): the structure presents as a hyperechoic object with a surrounding anechoic ring and shadow artifact deep field.

POCUS assessment of the lesion was completed using a Sonosite PX ultrasound machine with a 12MHz high-frequency linear transducer. A longitudinal sausage-shaped structure (8 x 12mm) was identified within the subcutaneous tissue which demonstrated a thin anechoic ring surrounding a hyperechoic structure with acoustic shadowing (Figure 1). There was no evidence of posterior acoustic enhancement that would have been anticipated if the lesion were a furuncle or cutaneous abscess. The structure was not compressible, nor did it appear to have any intrinsic movement.

**Figure 2:**
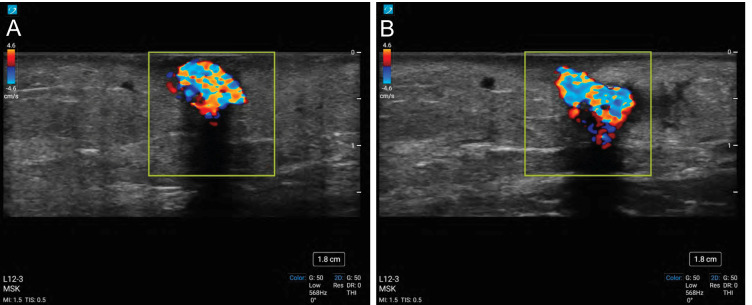
Twinkle artifact viewed withcolour Doppler scanning of the myiasis in short axis (A) and in long axis (B).

Interrogation of the lesion with colour Doppler was completed to assess if the structure had any vascularity prior to attempting an incision and excision. The lesion did not show any Doppler signal suggestive of vascular flow. Instead the structure, and continuing deep-field, demonstrated constant alternating colours of Doppler signal, giving the appearance of turbulent blood flow (Figure 2, Videos S1 & S2). This finding was consistent with a sonographic “twinkle sign”. 

**Figure 3:**
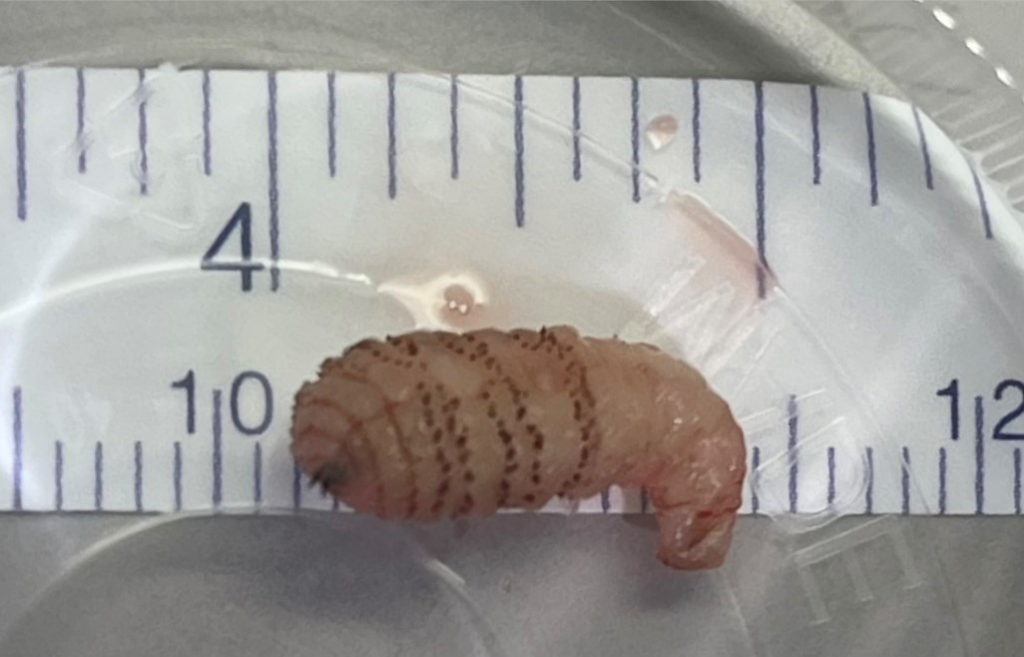
The *D. hominis* botfly assessed by POCUS, viewed after excision.

Following the POCUS examination, the area surrounding the foreign body was anesthetized with local anesthetic in preparation for an incision. Before the procedure could be completed, an organism was observed in the process of exiting the centre of the furuncle-type lesion. Traction with forceps succeeded in extracting an intact 12mm long organism (Figure 3). The patient was discharged home with no new prescription. Pathological examination subsequently confirmed that the specimen was a *D. hominis* larva (human botfly). During a follow-up phone call one month later, the patient reported complete resolution of his symptoms.

## Discussion

Previous reports on the use of formal ultrasound and POCUS to assess lesions caused by botfly myiasis have reported that the larvae appear as hyperechoic round or oval structures with shadow artifact 2, 3, 4. The larvae are usually noted to be surrounded by a ring of hypoechoic fluid 2, 4. Some authors have reported observing larval movement during the ultrasound scan 2, 3. The ultrasound findings in this case report are consistent with these observations, though we did not note any sonographic evidence of larval movement during our assessment. In contrast, POCUS assessment of a cutaneous abscess typically demonstrates contents that are anechoic to hyperechoic, with a hyperchoic rim that is hyperemic on colour doppler flow 5. Abscesses typically show posterior acoustic enhancement. Compression of an abscess can elicit sonographically visible swirling of the purulent contents 5. Application of colour doppler flow on an abscess would not be expected to show a twinkle artifact. 

The twinkle artifact (also known as twinkle sign or colour comet artifact) during Doppler ultrasound was first described in 1996 6. It presents as a rapidly changing mixture of red and blue signal deep field to a reflective structure and is understood to represent noise within the Doppler signal caused by reflections off of a highly echogenic surface 6. Clinicians classically look for a twinkle artifact during Doppler sonographic assessment for a calcium containing calculus (either nephrolithiasis or cholelithiasis). A retrospective chart review found that the presence of the twinkle sign on initial ultrasound had a high positive predictive value (78%) for the presence of nephrolithiasis on subsequent unenhanced CT 7. 

The twinkle sign has also been observed during sonography of other structures. Nagafuchi et al. reported on the presence of a twinkle artifact in rheumatologic patients when scanning periarticular calcification secondary to intra-articular corticosteroid injections 8. In a series of 46 consecutive patients with microcalcifications on mammogram, looking for the twinkle artifact during ultrasound-guided biopsies increased the sensitivity for suspicious lesions from a baseline of 30% with B-mode up to 89% with Doppler 9. The twinkle sign has also been described when scanning strongly reflective surfaces with a rough texture that do not contain any calcium, such as iron fillings and ground glass 6, 7. The artifact is not observed when scanning smooth reflective surfaces, such as metal wire 6.

Here we present the first known report documenting a twinkle artifact during the sonographic assessment of a myiasis. The etiology of the myiasis twinkle artifact is presumed to be a result of the hard, irregular and spiny larva carapace. It is possible that more immature larvae, with less developed carapaces, will not demonstrate twinkle artifact and that this sonographic characteristic may only be seen with later presentations of myiasis. This case involved a *D. hominis* larvae. Other species of myiasis (eg.* Cordylobia anthropophaga*) have a similar carapace composition and surface texture as *D. hominis*, and so we would anticipate that POCUS assessment of these lesions would produce a similar sonographic twinkle sign, but this is currently unknown 10. 

During our POCUS assessment the botfly larva was located within the patient’s subcutaneous tissues. Within a few minutes, however, the larva began spontaneously exiting the host. We suspect that the ultrasound gel applied during the POCUS exam acted as an occlusive coating, prompting the larva’s exit. In this case it is possible that in addition to diagnostic assistance, the POCUS provided some therapeutic value.

Due to the low incidence of botfly infestation in North America, patients with myiasis are often misdiagnosed with cellulitis 2, 3. This leads to unnecessary repeat healthcare visits, inappropriate use of antibiotics and a delay in the initiation of appropriate treatment. This report demonstrates the importance of maintaining a broad differential diagnosis for localized swelling and erythema, especially in the context of recent travel history, and the value of employing POCUS in order to make an accurate diagnosis. In this case, the application of POCUS demonstrated some features already described, as well as a newly described sonographic feature in the context of myiasis assessment, the myiasis twinkle sign.

## Statement of Consent

Informed consent was obtained for the use of the images in this report.

## Conflict of Interest

The authors have no conflicts of interest to declare.

## Supplementary Material

 Video S1 Video of the twinkle artifact viewed with colour Doppler scanning of the myiasis in short axis.

Video S2 Video of twinkle artifact viewed with colour Doppler scanning of the myiasis in long axis.
